# Improvement of isometric dorsiflexion protocol for assessment of tibialis anterior muscle strength^☆^

**DOI:** 10.1016/j.mex.2015.02.006

**Published:** 2015-02-18

**Authors:** Ariba Siddiqi, Sridhar P. Arjunan, Dinesh Kumar

**Affiliations:** Biosignals Lab Electrical and Computer Engineering, RMIT University, Melbourne, VIC 3000, Australia

**Keywords:** Improvement of isometric dorsiflexion protocol, Isometric dorsiflexion, Tibialis anterior, Triceps surae, Coactivation, Electromyogram

## Abstract

It is important to accurately estimate the electromyogram (EMG)/force relationship of triceps surae (TS) muscle for detecting strength deficit of tibalis anterior (TA) muscle. In literature, the protocol for recording EMG and force of dorsiflexion have been described, and the necessity for immobilizing the ankle has been explained. However, there is a significant variability of the results among researchers even though they report the fixation of the ankle.

We have determined that toe extension can cause significant variation in the dorsiflexion force and EMG of TS and this can occur despite following the current guidelines which require immobilizing the ankle. The results also show that there was a large increase in the variability of the force and the RMS of EMG of TS when the toes were not strapped compared with when they were strapped. Thus, with the current guidelines, where there are no instructions regarding the necessity of strapping the toes, the EMG/force relationship of TS could be incorrect and give an inaccurate assessment of the dorsiflexor TA strength.

In summary,

• Current methodology to estimate the dorsiflexor TA strength with respect to the TS activity, emphasizing on ankle immobilization is insufficient to prevent large variability in the measurements.
• Toe extension during dorsiflexion was found to be one source of variability in estimating the TA strength.
• It is recommended that guidelines for recording force and EMG from TA and TS muscles should require the strapping of the toes along with the need for immobilizing the ankle.

## Graphical abstract

A comparison of the existing isometric dorsiflexion protocol (Setting 1) and the proposed improvement of the protocol which includes the fixation of the toes in addition to fixing the ankle and foot (Setting 2) are shown. The triceps surae muscle group comprises of the lateral gastrocnemius (LG), medial gastrocnemius (MG) and soleus (SOL).

**Figure:**
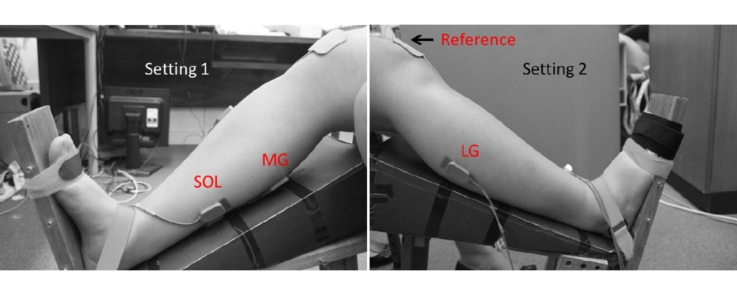

## Method details

### Isometric dorsiflexion protocol

Participants sit in a sturdy chair, with their leg extended to rest on a foot plate, such that their hip, knee and ankle are at 90°, 140°, and 90° angle, respectively. A force sensor is fixed to the foot plate and measures the force applied to it. The non-dominant foot of the subject is placed firmly on the ground, to prevent instability during isometric dorsiflexion.

• The ankle is secured to the foot plate with a flexible strap, such that no heel lift can occur.
• A flexible strap is secured at the junction of the metatarsal and phalanges, to ensure no foot movement can occur.
• A flexible strap is secured around the toe phalanges to secure the toes and prevent toe-extension.
• The participants are instructed to dorsiflex their ankle and the examiner ensures that there is no foot or toe movement.

## Method validation

To compare the proposed isometric dorsiflexion protocol with the existing method [1] that has been described in literature, isometric dorsiflexion force and amplitude of the muscle activity of the agonist tibialis anterior and antagonist triceps surae was recorded as follows:

Nine healthy participants (27.6 ± 5.7 years) with no prior neuromuscular injury or disease performed the isometric dorsiflexion protocol in two different positions: Position 1 – toes strapped, Position 2 – toes unstrapped. The experimental protocol was approved by RMIT University Human Research Ethics Committee and in accordance with Helsinki Declaration (revised 2004).

Electromyogram (EMG) is the recording of the muscle activity and in this study EMG was recorded from the tibialis anterior (TA) and the triceps surae muscle groups: medial gastrocnemius (MG), lateral gastrocnemius (LG) and soleus (SOL). Myomonitor 4 (Delsys, Boston) EMG data acquisition system was used. This has a gain of 1000, CMRR of 92 dB and bandwidth of 20–450 Hz, and 12 dB/octave roll-off. The sampling rate was 1000 Hz with a resolution of 16 bits/sample. Delsys single-channel active differential silver bar (10 mm × 1 mm) were used to record EMG. These have embedded preamplifiers and fixed inter-electrode distance of 10 mm. The skin at the electrode locations was abraded and cleaned with an alcohol swipe to reduce resistance, prior to electrode fixation. The electrode locations were determined from the SENIAM recommended positions as follows [2]:

TA: 1/3rd on the line between the tip of the fibula and the tip of the medial malleolus.

SOL: 2/3rd the line between the medial condylis of the femur to the medial malleolus.

MG: On the most prominent bulge of the muscle belly.

LG: 1/3rd the line between the head of fibula and the heel.

The force exerted during the dorsiflexion was recorded with a force sensor (Interface S type) that was attached to the foot plate.

Two different settings were used for fixing the foot of the participants to the foot plate: (i) following existing protocol, where the foot was fixed and ankle immobilized, and (ii) in addition to the current protocol, the toes were strapped. Participants were instructed to exert maximal voluntary contraction (MVC) for 5 s and repeated with a 120 s rest period. The higher of the two trials was recorded as the MVC of the individual. This was repeated for both the foot fixing protocols. Minimum 5 min rest period was given between two recordings. The first and the last 1 s of the recordings tended to have transients and thus was discarded. The root mean square (RMS) of the remaining 3 s of EMG recording were calculated for the TA and the three TS muscles: MG, LG and SOL. The RMS values of the TS muscles were summed to compute the overall TS RMS. Coefficient of variation of the force and TS EMG RMS was determined to investigate the change in variability.

The nonparametric Wilcoxon signed-rank test was performed to determine the statistical significance of the difference between the two settings. This test was chosen because of the small sample size.

**Fig. 1:**
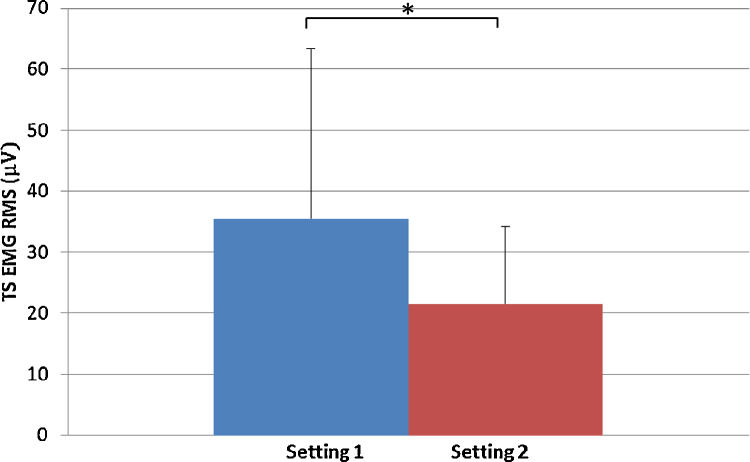
EMG RMS for the triceps surae muscle group for Setting 1 with toes unstrapped and Setting 2 toes strapped. *p < 0.05.

**Fig. 2:**
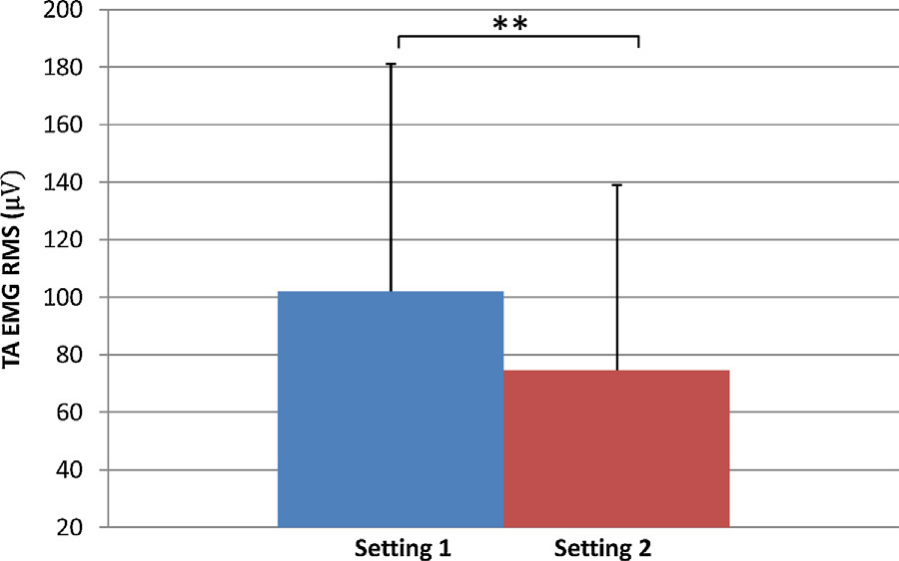
EMG RMS for the tibialis anterior for Setting 1 with toes unstrapped and Setting 2 toes strapped. **p < 0.1.

**Fig. 3:**
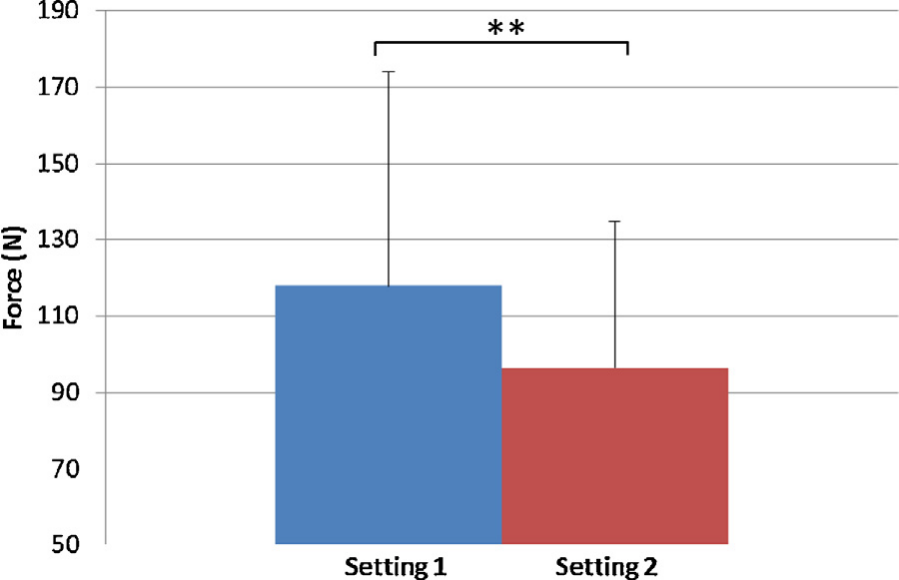
Dorsiflexion force (*N*) for Setting 1 with toes unstrapped and Setting 2 toes strapped. **p < 0.1.

The results show a 65% increase in the RMS of the triceps surae muscles in Setting 1 (35.5 ± 27.9 μV) compared with Setting 2 (21.5 ± 12.6 μV) (Fig. 1) while a 36% increase in the RMS of the TA was observed in Setting 1 (102.0 ± 79.1 μV) compared with Setting 2 (74.8 ± 64.3 μV) (Fig. 2). The results also show that the mean value of force (in Newton) was 22% higher in Setting 1 (117.9 ± 56.3 N), where the toes were unstrapped compared with Setting 2 where the toes were strapped (96.5 ± 38.4 N) (Fig. 3)

The coefficient of variation of the force and TS EMG activity were higher in Setting 1 (force: 47%, TS: 78%) in comparison to Setting 2 (force: 39% TS: 58%). This further supports our hypothesis that securing the toes during dorsiflexion would reduce the variability.

To determine if these results were repeatable, a second trial was performed by the participants. The nonparametric Wilcoxon signed-rank test determined no significant difference of the force exerted and the EMG activity of the TA and TS (*p* > 0.1) between the two trials.

## Additional information

There is a discrepancy in the reported age-related strength decline of the tibialis anterior (TA), with values ranging from 63% to 14% [3], [4], [5], [6], [7], [8], and some studies reporting that there is no significant decline [9], [10]. This discrepancy has been attributed to inaccurate assessment of the muscle strength in the protocols [1]. Taking into consideration the triceps surae (TS) antagonistic force, Simoneau et al. [1] found a non-significant decline in the resultant dorsiflexion MVC (−15%), but a significant decline in the agonist MVC during dorsiflexion (−39%). This finding asserts the importance of measuring the antagonist force for accurate measurements of muscle strength in the TA.

The force at the ankle is dependent on the joint angle because this changes the muscle length [11]. It also alters the contribution by the antagonist muscle to the resultant force [12], [13]. Thus, immobilizing the ankle during isometric plantar flexion or dorsiflexion to measure the force has been emphasized [14].

An example of the existing isometric dorsiflexion protocol is seen in Fig. 1 used by Simoneau et al. [1]. They have described the use of a shoe bolted on to a foot plate and a strap around the shoe to prevent heel lift or foot movement; however, they did not mention the need for fixation of the toes. As most shoes allow movement of toes, the singular strap would not have been sufficient in securing it, and hence the need for modifying the protocol to address this.

The action of toe extension leads to the tightening of the plantar fascia via the “windlass” mechanism [15]. It has been demonstrated that at higher dorsiflexion angles of the toes, greater Achilles tendon strains are transferred to the plantar fascia [16]. In converse, greater dorsiflexion angles of the toe leads to increased strain in the plantar fascia and consequently the Achilles tendon. This increased strain on the Achilles tendon may increase the dorsiflexion angle of the ankle, although this needs to be verified by ultrasound studies.

Coactivation of the agonist and antagonist muscle is used to stabilize the joint [17], distribute articular capsule pressure [18], and prevent bone displacements [19]. In fact, it has been shown that at higher dorsiflexion ankle angles, the coactivation level of the antagonist TS increased from the neutral ankle angle (90°) [20]. Therefore, the increased coactivation of the TS observed, could be due to an ankle joint instability due to toe extension. This increased coactivation would overestimate the agonist TA force, which is important for assessing muscular strength in the elderly.
